# Multilocus sequence typing and phenotypic properties of *Streptococcus mutans* from Thai children with different caries statuses

**DOI:** 10.1186/s12903-024-04759-9

**Published:** 2024-09-11

**Authors:** Jinthana Lapirattanakul, Ryota Nomura, Rena Okawa, Pornpen Tantivitayakul, Rattiporn Kaypetch, Anna Lehrkinder, Peter Lingström, Dowen Birkhed, Michiyo Matsumoto-Nakano, Kazuhiko Nakano

**Affiliations:** 1 Department of Oral Microbiology, Faculty of Dentistry, Mahidol University, 6 Yothi Street, Rajthevi, Bangkok, 10400 Thailand; 2 Department of Pediatric Dentistry, Osaka University Graduate School of Dentistry, Osaka, Japan; 3 Department of Pediatric Dentistry, Graduate School of Biomedical and Health Sciences, Hiroshima University, Hiroshima, Japan; 4 Research Office, Faculty of Dentistry, Mahidol University, Bangkok, Thailand; 5 Department of Cariology, Institute of Odontology, Sahlgrenska Academy, University of Gothenburg, Gothenburg, Sweden; 6 Fersens väg 14B, Malmö, 211 42 Sweden; 7 Department of Pediatric Dentistry, Okayama University Graduate School of Medicine, Dentistry, and Pharmaceutical Sciences, Okayama, Japan

**Keywords:** Acid production, Acid tolerance, Biofilm formation, Caries status, Collagen binding, Multilocus sequence typing, *Streptococcus mutans*

## Abstract

**Background:**

*Streptococcus mutans* is studied for its acidogenic and aciduric characteristics, notably its biofilm formation in the presence of sucrose, toward its role in the caries process. Variations in both genotype and phenotype have been reported among clinical isolates of *S. mutans*. This study aimed to examine genotypic and phenotypic characteristics of *S. mutans* obtained from Thai children with varying caries statuses.

**Methods:**

We determined the presence of *S. mutans* and caries status in 395 children aged 3–4 years. From 325 children carrying *S. mutans*, we selected 90 with different caries statuses—caries-free (CF; *n* = 30), low severity of caries (LC; *n* = 30), or high severity of caries (HC; *n* = 30). Three isolates of *S. mutans* were taken from each child, thus, a total of 270 isolates were obtained. Multilocus sequence typing (MLST) was used to genotype the isolates and assess their clonal relationships. The properties, including biofilm formation, collagen binding, and acid production and tolerance were also evaluated.

**Results:**

Children with carious lesions showed a higher detection rate and number of *S. mutans* in saliva than those without caries. *S. mutans* from individuals with HC status showed the lowest biofilm formation ability, while this group had the highest detection rate of collagen-binding isolates. There was no difference in acid production or tolerance by caries status. Genotyping by MLST did not reveal any clone of *S. mutans* specific to CF status. This result remained even when we included MLST data from the open-access PubMLST database. MLST did identify clones containing only strains from caries-affected hosts, but tests of their phenotypic properties did not reveal any differences between *S. mutans* from these clones and clones that were from both caries-free and caries-affected children.

**Conclusions:**

The clonal relationships of *S. mutans* indicated by MLST were not associated with the status of dental caries in the host.

**Supplementary Information:**

The online version contains supplementary material available at 10.1186/s12903-024-04759-9.

## Introduction

Dental caries is a biofilm-mediated, diet-modulated, multifactorial disease where microbes also play a role in its occurrence [1]. Carious lesions arise from an imbalance in the oral microbiota, leading to dysbiosis and the formation of an acidic environment near tooth surfaces. Rather than being caused by a specific pathogen, the caries process involves the interplay of diverse species of microorganisms, including a well-known species, *Streptococcus mutans*, which was first isolated from a carious lesion [2–4]. The characteristics of *S. mutans*, studied for its role in the caries process, include the ability to efficiently colonize tooth surfaces (adhesion), along with acid production and tolerance [5]. These properties have also been investigated and demonstrated in other oral microbes associated with dental caries [6–8].

Although *S. mutans* has been found with a high prevalence in humans with carious lesions, this bacterium has also been reported in the oral cavities of caries-free individuals [4, 9, 10]. Various methodologies have confirmed variability in both genotypic and phenotypic characteristics of clinical isolates of *S. mutans* [11–13]. Consequently, some researchers have explored the differences between *S. mutans* strains from caries-free and caries-active hosts [11, 14–17]. Nevertheless, changes in microbial compositions and properties are also influenced by factors such as dietary habits, oral hygiene, and environmental changes during caries formation [7, 18, 19].

Multilocus sequence typing (MLST) has been proposed as a reproducible method to characterize and identify clonal relationships of bacteria [20]. Because it is a molecular typing method based on the nucleotide sequences of multiple housekeeping genes, MLST creates a standardized approach for gathering and comparing data via web-based databases. PubMLST (https://pubmlst.org/) is a website dedicated to the collection of open-access and curated MLST data [21]. PubMLST provides sequence data with provenance and phenotype information for > 130 microbial species and genera. MLST has been used widely to distinguish genotypes and study both prokaryotic and eukaryotic microorganisms in the oral cavity, including the population structure of microbes [22, 23] and to identify potential disease-related clones or clones associated with virulence factors and antimicrobial resistance traits [24–26]. Beginning in 2007, our research group has developed an MLST scheme for *S. mutans* [12]. This scheme was advantageous in specifying *S. mutans* clones with particular serotypes and virulence potential regarding bacteremia and infective endocarditis [27–29].

In the present study, we used this MLST scheme to genotype *S. mutans* sampled from the oral cavity of Thai children and to study their clonal relationships. We undertook genotypic study of these *S. mutans* isolates, and examined their phenotypes, i.e., biofilm formation in the presence of sucrose, ability to bind with collagen, acid production, and acid tolerance. Additionally, we supplemented our MLST results with those deposited in the PubMLST database regarding caries. We aimed to find whether there were associations of genotypic and phenotypic characteristics of *S. mutans* with the status of dental caries in the host.

## Materials and Methods

### Subjects, saliva collection, and dental examination

Three hundred and ninety-five subjects (216 boys and 179 girls), 3–4 years of age, with no prior history of antibiotic use, were recruited from a kindergarten in Bangkok, Thailand. Samples were collected between 8: 30 and 10: 30 a.m. Participants were instructed not to brush their teeth before collection. Saliva was obtained by asking participants to expectorate 2–3 ml of saliva into a sterile 50-ml tube. The saliva was kept on ice for ≤ 3 h before bacterial isolation.

Data on caries experience were recorded by the decayed, missing, filled teeth/surfaces (dmft/s) indices based on modified World Health Organization (WHO) criteria, which cavitated lesions within the enamel were also included as part of decayed teeth and decayed surfaces. In addition, the numbers of decayed teeth and decayed surfaces were reported as dt and ds, respectively.

Oral hygiene status was assessed by the Silness and Löe plaque index, which records the amount of plaque (score 0–3) on six teeth (maxillary right second molar, maxillary right lateral incisor, maxillary left first molar, mandibular right first molar, mandibular left lateral incisor, and mandibular left second molar) [30]. The average of all scores from these teeth was presented as the plaque index for each child.

Some subjects in the present study were also studied and presented in Lapirattanakul et al. [10]. The research protocol for the present study was approved by the Ethics Committee of Mahidol University (MU-DT/PY-IRB 2015/DT048; COA.No.MU-DT/PY-IRB 2015/040.0109), and prior written informed consent was obtained from the parents or legal guardians of all participants.

### Determination of numbers of *S. mutans*

The collected saliva specimens were tenfold serially diluted in saline solution and cultured on Mitis Salivarius agar (Difco Laboratories, Detroit, MI, USA) containing bacitracin (0.2 U/ml; Sigma Chemical Co., St. Louis, MO, USA), 0.001% (v/v) tellurite solution (Becton, Dickinson and Co., Sparks, MD, USA) and 15% (w/v) sucrose (MSB agar plates). After a 2-day incubation at 37 °C in a humidified atmosphere containing 5% CO_2_, the number of *S. mutans* was detected from MSB agar plates based on colony morphology (raised, convex, undulate, opaque, pale-blue colonies that are granular or frosted glass in appearance. Colonies may exhibit a glistening bubble on the surface due to glucan synthesis). Then, the number of *S. mutans* in the saliva samples was enumerated in colony-forming units (CFU) per milliliter of saliva.

### Isolation of *S. mutans* from Thai children with different caries statuses

Based on microbiological data, we identified children carrying *S. mutans*, and through dental examination, we classified them into three groups: (i) caries-free (CF), (ii) low severity of caries (LC), or (iii) high severity of caries (HC). We then randomly selected 30 children from each group. The 30 children in the CF group were caries-free and had no experience with dental caries. The LC group contained 30 children with fewer than five decayed teeth (dt < 5), and the HC group was composed of 30 children having at least 10 decayed teeth (dt ≥ 10). The carious lesions in the LC group were limited to enamel breakdown without visible dentin. In contrast, the decayed teeth in the HC group also contained dentin caries.

Three colonies of *S. mutans* were randomly picked from each subject’s MSB agar plate based on colony morphology. Thus, collectively, 270 isolates of *S. mutans* were obtained for further evaluation. All *S. mutans* isolates were kept as stocks in brain heart infusion (BHI) broth containing 50% glycerol at − 80 °C.

### Biofilm formation assay

Biofilm formation in the presence of sucrose was evaluated in 96-well flat-bottom plates (Thermo Fisher Scientific, Jiangsu, China). The procedure followed that in our previous publication with some modifications [31]. One-half strength Todd-Hewitt (TH) medium (Becton, Dickinson and Co.) with 0.25% sucrose was used as the culture medium. Pre-grown *S. mutans* cultured at 37 °C for 16–18 h were diluted 1:100 in the medium, then distributed at 100 μl per well. The plates were then incubated at 37 °C under 5% CO_2_ for 24 h. After discarding the medium from the wells, the plates were washed three times with distilled water. Formed biofilms were fixed with 100 µl of 25% formaldehyde at room temperature for 10 min. Following washing with distilled water, biofilms were then stained with 100 µl of 0.05% (w/v) crystal violet in water for 1 min, and washed with water. The absorbance at 590 nm (A_590 nm_) of the dye dissolved in 100 µl of 7% acetic acid was measured using an ELx800TM Absorbance Reader (BioTek Instruments Inc., Winooski, VT, USA). Three independent experiments were performed in triplicate.

### Collagen-binding assay

Collagen-binding properties of *S. mutans* isolates were determined by a previously described procedure with some modifications [28]. In brief, 10 µg of type I collagen in 0.1 M acetic acid (Sigma) was coated onto each well of a 96-well (round-bottom) cell culture plate (Corning Incorporated, Corning, NY, USA) and incubated at 4 °C for 24 h. Plates were washed with phosphate-buffered saline (PBS) and incubated with 5% bovine serum albumin (Sigma) in PBS at 37 °C for 1.5 h, washed with PBS containing 0.01% Tween 20, and then 1 × 10^9^ *S. mutans* cells in PBS were added to each well. After 1 h of incubation at 37 °C, adherent cells were washed with PBS, and treated with 25% formaldehyde, 0.05% crystal violet, and 7% acetic acid as described above in the section “Biofilm formation assay”. The collagen-binding ability of the tested isolates is presented as A_590 nm_ of the crystal violet dye in 7% acetic acid measured by using the ELx800TM Absorbance Reader. *S. mutans* strain TLJ11–2 possessing collagen-binding ability and *S. mutans* strain TLJ1-1 lacking this ability [27] were used as positive and negative controls, respectively. Three independent experiments were conducted in triplicate.

### Acid production assay

To compare acid production among the *S. mutans* isolates, an acid production assay was performed using our previously reported method [31]. Two milliliters of a seed culture of *S. mutans* (10^9^ CFU/ml) were inoculated into 200 ml of phenol red broth (Difco) containing 1% glucose. The cultures were incubated in closed containers at 37 °C for 24 h. During incubation, 2 ml of the culture mixture was collected at 0, 4, 8, 12, 18, and 24 h, and the pH was determined using a pH meter (Ohaus Corporation, NJ, USA). Three independent experiments were performed for all tested *S. mutans*.

### Acid tolerance assay

An acid tolerance assay based on our previous report was used [31]. *S. mutans* were cultured overnight in TH broth containing 0.3% yeast extract (THYE), then diluted tenfold in fresh THYE and incubated at 37 °C until reaching the mid-logarithmic growth phase (OD_600nm_ = 0.4–0.5). Bacterial cells were harvested and resuspended in THYE (pH 5.0), then incubated at 37 °C for 2 h. Acid tolerance was detected by incubating the cells at 37 °C at a potential killing pH of 3.5 for 2 h. Cells were counted in triplicate by plating on THYE plates before and after incubation at the killing pH. Results are shown as the percentage survival rate, which was calculated using the formula: 100 × (number of cells following incubation at pH 3.5)/(number of cells before incubation at pH 3.5).

### MLST analysis of *S. mutans*

*S. mutans* isolates were genotyped by our previous MLST scheme with some modifications [12]. Firstly, genomic DNA was extracted by the protocol for Gram-positive bacteria [27]. The obtained DNA was confirmed to be *S. mutans* genome using PCR with species-specific primers [32]. Then, internal fragments of eight housekeeping genes, namely, transketolase (*tkt*), glutamine synthetase type I (*glnA*), glutamate synthetase (*gltA*), glucose kinase (*glk*), shikimate 5-dehydrogenase (*aroE*), glutamate racemase (*murI*), signal peptidase I (*lepC*), and DNA gyrase A subunit (*gyrA*), were PCR amplified using primers shown in Table S1. The reaction mixture (20 µl) contained 0.5 U Ex *Taq*™ DNA polymerase (Takara Bio Inc., Shiga, Japan), 2 µl of 10 × Ex *Taq* buffer (Takara Bio) containing 20 mM Mg^2+^, 1.6 µl dNTPs (2.5 mM), 0.5 µl each of primer (20 µM), 40 ng DNA, and 13.3 µl sterilized water. Thermocycling was carried out in a Bioer Life Express thermocycler (Bioer, Hangzhou, China) as follows: 94 °C for 5 min; followed by 25 cycles (30 cycles for *tkt*) at 94 °C for 30 s, 55 °C (50 °C for *tkt*) for 30 s, and 72 °C for 30 s; with a final extension at 72 °C for 7 min. After checking PCR products by agarose gel electrophoresis visualized by staining with ethidium bromide, the products were purified with illustra ExoProStar 1-Step (GE Healthcare, Little Chalfont, UK), and sequenced (Macrogen Inc., Seoul, South Korea) using their respective PCR primers. All nucleotide sequences from these isolates determined in the process of MLST were registered in the GenBank database (accession numbers OQ809072–OQ809341 for *tkt*; OQ829640–OQ829909 for *glnA*; OQ829910–OQ830179 for *gltA*; OQ830180–OQ830449 for *glk*; OQ866634–OQ866903 for *aroE*; OQ938982–OQ939251 for *murI*; OQ939252–OQ939521 for *lepC*; and OQ972993–OQ973262 for *gyrA*).

Distinct nucleotide sequences in each housekeeping locus were assigned different allele numbers. For an *S. mutans* isolate, the allele numbers for each of the eight loci define the allelic profile and consequently the sequence type (ST). There is a public database gathering information on *S. mutans* studied by MLST; hence the nucleotide sequences of housekeeping gene fragments obtained in this study were compared with those deposited in the Oral *Streptococcus* PubMLST database (http://pubmlst.org/oralstrep/) [21]. The same allele numbers and STs were assigned for matched results, while new ones were submitted to the database for designation.

Allelic profiles of the 270 *S. mutans* isolates were analyzed using START (sequence type analysis and recombinational tests) [33] to produce a matrix of pairwise differences in the allelic profiles, and a dendrogram was constructed from the matrix using the unweighted pair group method with arithmetic mean. Related STs were grouped by using the goeBURST (global optimal enhanced based upon related sequence types) algorithm implemented in the PHYLOViZ program [34]. Two or more independent isolates sharing identical alleles at ≥ 6 loci were defined as members of a ‘clonal complex’ (CC) or ‘group’ of STs that have diversified from a common ancestor.

Moreover, the allelic profiles of the 270 *S. mutans* in the present study were further analyzed for relatedness together with profiles of 115 strains selected from the Oral *Streptococcus* PubMLST database (Table S2). The selection criteria were that these 115 strains of *S. mutans* were genotyped by the same MLST scheme as used in the present study (the Nakano scheme) [12], and that the strains had precise information regarding the caries status of the subject from whom they were isolated [35–40].

#### Statistical analysis

SPSS software v.18.0 (SPSS Corp., Chicago, IL, USA) and GraphPad Prism software v.5.0 (GraphPad Software Inc., La Jolla, CA, USA) were used in statistical analyses. The χ^2^ test was used to test association for categorical variables. Student’s *t*-test, the Mann–Whitney U test (Mann-U), and the Kruskal–Wallis test were used to compare continuous variables. Correlation analysis was performed by using Spearman’s rank correlation. The level of significance was set at *p-*value < 0.05.

## Results

### Dental examination and microbiological data

Among 395 kindergarten children recruited in Bangkok, Thailand, 284 had decayed teeth (71.9%); 71.3% of boys and 72.6% of girls had carious lesions (no statistically significant difference by sex). The group with dental caries had a higher plaque index compared to the group without caries (*p*-value < 0.001, Mann-U test) (Table 1). *S. mutans* was detected in 325 children (82.3%). The detection rate of this bacterium was higher in caries-affected children than in those with no caries (*p*-value < 0.001, χ^2^ test). In the subjects carrying *S. mutans*, the value of log CFU/ml of *S. mutans* was higher in the saliva of caries-affected children than in children without caries (*p*-value < 0.001, *t*-test). In addition, a positive correlation was found between log CFU/ml of *S. mutans* and all caries indices (*p*-value < 0.001, Spearman’s rank correlation); the correlation coefficients (r_s_) were 0.475 (dmft), 0.478 (dmfs), 0.488 (dt), and 0.493 (ds).

**Table 1:** Clinical and microbiological data for participating kindergarten children from Bangkok, Thailand

Subjects	Plaque index	Caries index				Subjects with *S. mutans*	Log CFU/ml of *S. mutans* in saliva
		dt	dmft	ds	dmfs		
Without caries (*n* = 111)	0.56 ± 0.38^a^ (0.50)	0 ± 0 (0)	0 ± 1 (0)	0 ± 0 (0)	1 ± 3 (0)	63 (56.8)^b^	4.05 ± 1.20^c^ (4.00)
With caries (*n* = 284)	0.99 ± 0.47^a^ (1.00)	7 ± 4 (6)	7 ± 5 (6)	13 ± 13 (8)	15 ± 14 (10)	262 (92.3)^b^	5.12 ± 1.02^c^ (5.18)
Total (*n* = 395)	0.87 ± 0.47 (0.83)	5 ± 5 (4)	5 ± 5 (4)	9 ± 12 (4)	11 ± 14 (5)	325 (82.3)	4.92 ± 1.14 (5.04)

Data are presented as the mean ± *SD* (median), or *n* (% within caries status of subjects). *dt* number of decayed teeth, dmft, number of decayed, missing, and filled teeth, *ds* number of decayed tooth surfaces, *dmfs* number of decayed, missing, and filled tooth surfaces

^a^*p*-value < 0.001, Mann–Whitney U test

^b^*p*-value < 0.001, χ^2^ test

^c^*p*-value < 0.001, *t*-test

Data regarding the plaque index, the caries indices, and the number of bacteria in the saliva of the 90 children who were the sources of the 270 *S. mutans* isolates analyzed further in this study are shown in Table 2; the children were divided into three caries statuses: caries-free (CF), low severity of caries (LC), and high severity of caries (HC).

**Table 2:** Clinical and microbiological data for 90 children classified into three groups by caries status

Groups	Plaque index	Caries index				Log CFU/ml of *S. mutans* in saliva	Subjects with mixed STs of *S. mutans*
		dt	dmft	ds	dmfs		
Caries-free (*n* = 30)	0.39 ± 0.29 (0.42)	0 ± 0 (0)	0 ± 0 (0)	0 ± 0 (0)	0 ± 0 (0)	4.10 ± 1.04 (4.10)	9 (30.0)
Low severity of caries (*n* = 30)	0.82 ± 0.42 (0.83)	2 ± 1 (2)	2 ± 1 (2)	2 ± 1 (2)	3 ± 2 (2)	4.66 ± 1.12 (4.83)	6 (20.0)
High severity of caries (*n* = 30)	1.16 ± 0.49 (1.09)	13 ± 3 (13)	14 ± 3 (13)	32 ± 14 (30)	34 ± 16 (31)	5.75 ± 0.78 (5.68)	13 (43.3)
*p*-value	< 0.001^a^					< 0.001^a^	0.147^b^

Data are presented as the mean ± *SD* (median), or *n* (% within caries status of subjects) *dt* number of decayed teeth, *dmft* number of decayed, missing, and filled teeth, *ds* number of decayed tooth surfaces, *dmfs* number of decayed, missing, and filled tooth surfaces, *ST* sequence type

^a^Kruskal-Wallis test

^b^χ^2^ test

### Biofilm formation and collagen binding of *S. mutans* from children with the three caries statuses

**Fig. 1:**
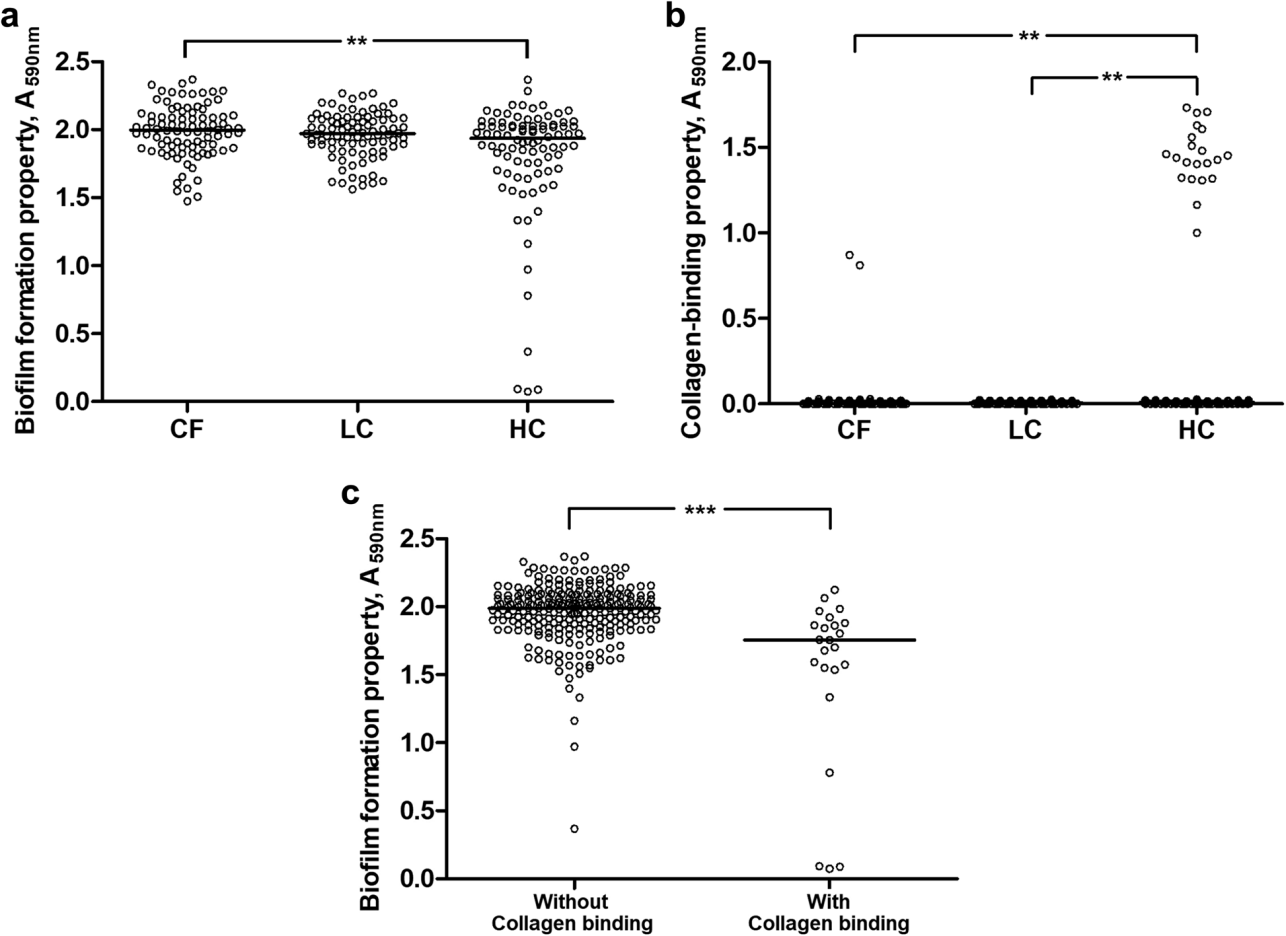
**a** Biofilm formation and **b** collagen-binding properties of *S. mutans* from Thai children. The comparison was based on caries status of the donor: caries-free (CF; *n* = 90 *S. mutans* isolates), low severity of caries (LC; *n* = 90), and high severity of caries (HC; *n* = 90). *S. mutans* from children in the HC group showed the lowest biofilm formation ability and the highest collagen-binding ability. **p-value < 0.01, Kruskal–Wallis test. **c** In addition, *S. mutans* with collagen-binding ability (*n* = 23) showed lower biofilm formation ability than the non-collagen binding isolates (*n* = 247). ***p-value < 0.001, Mann–Whitney U test

All 270 isolates of *S. mutans* could form biofilm in a culture medium with sucrose. The biofilm formation, represented by the A_590 nm_ value, ranged from 0.073 to 2.369. When the ability to form biofilm in the presence of sucrose was compared based on the caries status of the host child, *S. mutans* isolated from children in the HC group showed the lowest biofilm formation (*p*-value < 0.01, Kruskal–Wallis test). In addition, isolates with low biofilm formation properties, indicated by A_590 nm_ < 1.500, were mostly present in the HC group (Fig. 1a).

The collagen binding assay showed that only 23 of the 270 *S. mutans* isolates could bind to collagen (for which A_590 nm_ ranged from 0.810 to 1.733) (Fig. 1b). These 23 isolates belonged to subjects in the HC (21 isolates) and CF (2 isolates) groups, thus there was an association between having collagen-binding ability and the caries status of the host (*p*-value < 0.001, χ^2^ test). Interestingly, *S. mutans* with collagen-binding ability showed lower biofilm formation than those without collagen-binding ability (*p*-value < 0.001, Mann-U test) (Fig. 1c).

### Acid production and acid tolerance of *S. mutans* from children with the three caries statuses

Acid production in the presence of 1% glucose showed no significant difference considering pH values at any time point (0 to 24 h) among *S. mutans* from subjects with different caries statuses (Fig. S1). Moreover, acid tolerance was similar among the three groups analyzed; there was no difference in the percentage of surviving cells after treatment in an acid environment (median survival: CF = 43.18%, LC = 43.09%, HC = 42.41%; *p*-value = 0.208, Kruskal–Wallis test).

### Genotyping of *S. mutans* isolates by MLST

A total of 270 *S. mutans* isolates from 90 children were classified into 81 unique STs, of which ST2 was the most common (Table S3). In 62 children (68.9%), the same ST was detected for all three isolates from the child. For the other 28 children (31.1%), MLST indicated mixed STs, i.e., two STs were found in 27 children, and three STs in one child (the child in HC status). The right-hand column of Table 2 shows the number of children with mixed STs of *S. mutans* in the CF, LC, and HC groups. No significant difference in the percentage of subjects with mixed STs was found according to caries status (*p*-value = 0.147, χ^2^ test).

### Clonal relationships of *S. mutans* analyzed by MLST

**Fig. 2:**
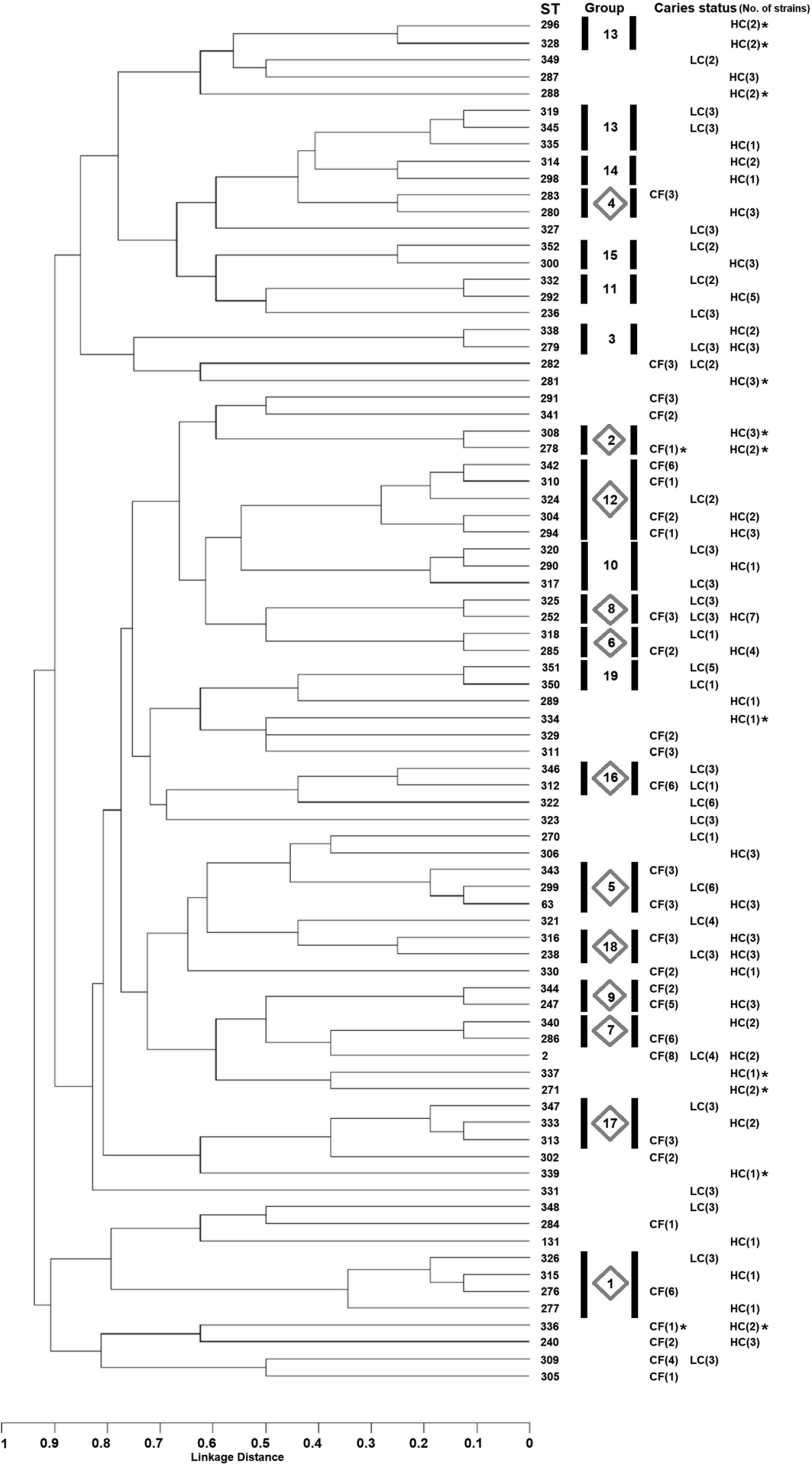
Dendrogram of 270 *S. mutans* isolated from Thai children with different caries statuses, i.e., caries-free (CF; *n* = 90 *S. mutans* isolates), low severity of caries (LC; *n* = 90), or high severity of caries (HC; *n* = 90). The dendrogram was constructed from allelic profiles of 81 sequence types (STs) detected by multilocus sequence typing (MLST). MLST indicated 30, 31, and 39 STs from individuals with CF, LC, and HC status, respectively. Clonal complex groups and the number of strains in each ST are indicated based on caries status. Asterisks indicate *S. mutans* with collagen-binding ability. The gray diamond (◇) indicates a clonal complex group containing *S. mutans* from both caries-free and caries-affected hosts

Determination of the clonal relationships of the 81 STs identified from the 270 *S. mutans* isolates revealed 32 STs as singletons unrelated to any other ST. The remaining 49 STs were grouped into 19 CCs (Fig. 2). No CC contained only strains from CF status individuals—the 12 CCs possessing *S. mutans* strains from CF subjects also contained strains from LC and HC status donors (Groups 1, 2, 4, 5, 6, 7, 8, 9, 12, 16, 17, and 18). The remaining seven CCs consisted of *S. mutans* from caries-affected subjects only (Groups 3, 10, 11, 13, 14, 15, and 19). Group 14 consisted of only *S. mutans* from children with HC status, while only strains from those with LC status were present in Group 19. The number of STs in most of these seven CCs was two, but Groups 10 and 13 contained three and five STs respectively. Considering collagen binding, Groups 2 and 13 contained 100% and 40% STs with collagen-binding ability (Fig. 2). No CC showed an association with ability to form biofilm or production or tolerance of acid. *S. mutans* in the seven CCs consisting only of *S. mutans* from caries-affected hosts showed no difference in phenotypic properties (biofilm formation, collagen binding, acidogenicity, and acid tolerance) from those in the 12 CCs that included strains from hosts with and without dental caries (Table 3).

**Table 3:** Comparisons of phenotypic properties of *S. mutans* based on types of clonal complex

*S. mutans* in	Biofilm formation property (A_590_)	Collagen-binding property (A_590_)	pH value at 24 h	Acid tolerance (%)
Groups^a^ 1, 2, 4, 5, 6, 7, 8, 9, 12, 16, 17, and 18 (*n* = 126)	1.96 ± 0.22 (1.99)	0.067 ± 0.281 (0.004)	4.44 ± 0.37 (4.35)	42.11 ± 2.27 (42.52)
Groups^b^ 3, 10, 11, 13, 14, 15, and 19 (*n* = 47)	1.89 ± 0.29 (1.97)	0.128 ± 0.401 (0.005)	4.50 ± 0.31 (4.37)	41.66 ± 2.68 (41.33)
*p*-value^c^	0.464	0.316	0.520	0.426

Data are presented as the mean ± *SD* (median)

^a^Twelve clonal complex (CC) groups containing *S. mutans* from both caries-free and caries-affected children

^b^Seven CC groups containing *S. mutans* from caries-affected children only

^c^Mann-Whitney U test

When we included the data for 115 strains from the Oral *Streptococcus* PubMLST database in the evaluation of clonal relationships, the number of CCs increased to 36 (Table 4). The largest CC was Group 5, having 14 STs, with China, Sweden, Thailand, and the USA as the countries of origin. ST2, the most common ST among Thai children in the present study, was located in this CC (Table S3). From a total of 36 CCs, there were 20 CCs containing *S. mutans* from both caries-free and caries-affected hosts. We still did not find a CC related to *S. mutans* from caries-free individuals only.

**Table 4:**
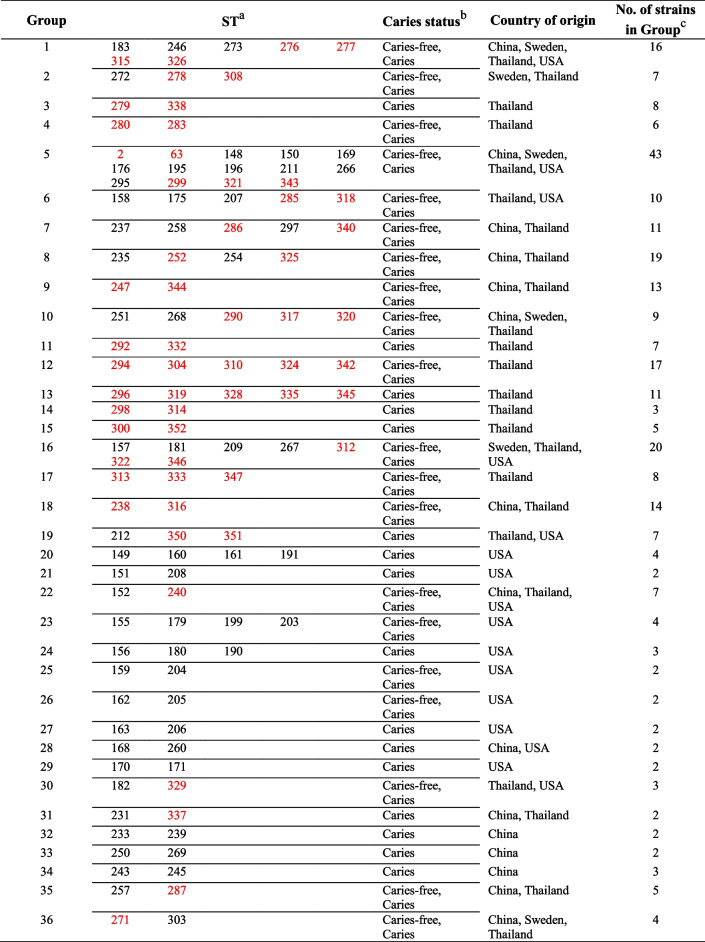
Clonal complex groups by goeBURST analysis of *S. mutans* isolated from Thai children together with *S. mutans* strains from the Oral *Streptococcus* PubMLST database

^a^ST (sequence type) is defined by the nucleotide sequences of eight housekeeping genes based on MLST analysis. Red letters indicate the STs containing *S. mutans* isolated from Thai children in this study

^b^Low severity of caries (LC) and high severity of caries (HC) in Thai children are grouped under “Caries” status

^c^Total number of *S. mutans* strains in each clonal complex group

## Discussion

The caries prevalence of Thai children in the present study was 71.9%. The plaque index indicated that the oral hygiene status of children with caries was poorer than that of children without caries. Additionally, children with carious lesions showed a higher detection rate and number of *S. mutans* in their saliva than those without caries. This can be attributed to the fact that individuals with poorer oral hygiene and more plaque tend to develop higher levels of *S. mutans* in their saliva compared to children with less plaque. Our findings are consistent with previous reports showing the association of *S. mutans* with the cariogenic environment and poor oral hygiene [9, 19, 41]. In the children carrying *S. mutans*, we found a positive correlation between the number of salivary *S. mutans* and caries indices, though the correlation coefficients indicated only weak correlations (r_s_ < 0.5). Notably, *S. mutans* was detected in more than 50% of children without caries. This is reasonable given the multifactorial etiology of dental caries as well as evidence suggesting the role of acidogenic and aciduric bacteria other than *S. mutans* in caries occurrence [2, 7]. Moreover, the presence of acidogenic and aciduric microorganisms alone cannot predict caries risk without the influence of a diet rich in sugars and frequent sugar consumption [18, 42].

Our study also evaluated the phenotypic traits of *S. mutans* from children with one of three caries statuses: CF, LC, or HC. Statistically significant differences were observed among the *S. mutans* sampled from these groups in terms of biofilm formation in the presence of sucrose and collagen binding. *S. mutans* from children having ≥ 10 decayed teeth, i.e., the HC group, showed the lowest biofilm formation ability, while the detection rate of *S. mutans* with collagen-binding ability was highest in this group. Either poor oral hygiene with a high amount of accumulated plaque or the high number of carious lesions in this HC group might help retain strains with different binding efficiencies and mechanisms. Therefore, *S. mutans* in the HC group exhibited a greater variety of biofilm formation in the presence of sucrose compared to both the CF and LC groups.

Both biofilm formation and collagen binding are related to the adhesion of, and colonization by, *S. mutans* [43]. All *S. mutans* in the present study could form biofilm in the medium containing sucrose, however, binding to collagen was not ubiquitous in our *S. mutans* isolates, approximately 10% of the evaluated *S. mutans* bound to type I collagen. This percentage was consistent with the previous findings [27]. Almost all *S. mutans* having collagen-binding ability were isolated from children with HC status, suggesting a potential link between collagen-binding ability, plaque accumulation, dentin access, and caries severity, though it did not establish causation. Some works have suggested an association between *S. mutans* strains with collagen-binding proteins and an increase in caries parameters and caries risk [44, 45]. In dentin caries, the ability to adhere to collagen may endow *S. mutans* with an alternative to the sucrose-dependent mechanism of colonization [43]. However, the risk of developing caries involves multiple factors beyond the severity and damage to the dentin. Fig. 1c showed that *S. mutans* with collagen-binding properties formed less monospecies biofilm in the presence of sucrose than the non-collagen binding isolates; thus, binding to collagen seemed to compensate for the low ability to form biofilm. In addition, the carious lesions exposing dentin collagen in the HC group might influence the high detection rate of collagen-binding *S. mutans* in this group.

In contrast to the biofilm formation and collagen-binding properties, the acidogenic and aciduric properties of *S. mutans* isolates were similar irrespective of the caries status of the host. *S. mutans* from donors with all three caries statuses (CF, LC, and HC) could decrease pH to < 5.5, the critical pH of enamel, within the experimental period, and nearly half of the vital cells of the isolates from all three statuses could survive at pH 3.5. This demonstrates the bacterium’s ability to thrive in acidic environments, highlighting its adaptability in conditions that contribute to the caries process. In multiple previous studies of acidogenic and aciduric properties of *S. mutans*, only some detected differences that were dependent on caries statuses [11, 14, 16, 17, 46–48]. Variations in both methodology and culture medium used can lead to such inconsistent findings. A study showed that the acid production in BHI broth was significantly greater for *S. mutans* from subjects with caries compared with those without caries, but this difference was not observed when a chemically defined medium was used [17].

For genotyping study of the *S. mutans* isolates, we used the allele-based MLST approach, which considers the allele as the unit of analysis [12]. Nucleotide sequence data were compared for each housekeeping locus. Distinct sequences were assigned different allele numbers. Because eight housekeeping genes are included in this MLST scheme, eight allele numbers define the allelic profile and the ST of the *S. mutans* isolate. The detection of the same ST among analyzed *S. mutans* isolates indicates that they are identical. In our present study, the genotyping of 270 *S. mutans* isolates by MLST found 81 STs. Because these *S. mutans* were from 90 children, we analyzed the number of STs in each child. In a majority of subjects (68.9%), the three isolates from the subject were classified in the same ST. These findings were consistent with results using the same MLST scheme to genotype *S. mutans* from Japanese and Chinese populations [39, 49]. Previous studies have evaluated whether there is an association between the number of *S. mutans* genotypes and caries activity. Most studies indicated more genotypes of *S. mutans* in children having severe caries compared with those who were caries-free [14, 37, 50, 51], but other authors found the opposite result [52], or no difference in genotypic diversity among children with different caries statuses [11, 16]. Certainly, the typing method used and the number of isolates analyzed could influence the results. In the present study, the children carrying multiple *S. mutans* STs were mostly found in the HC group, and one child in this group showed three STs. Thus, the genotypic diversity of *S. mutans* seemed higher in the HC group compared with the CF and LC groups. However, there was no statistically significant difference; thus, analyzing only three isolates of *S. mutans* per subject might limit statistical findings in this aspect.

The MLST scheme used for *S. mutans* in this study was based on eight housekeeping genes [12]. In analysis of relatedness among the analyzed strains, two or more isolates of *S. mutans* sharing identical alleles at least six housekeeping loci were grouped into a CC of STs that have diversified from a common ancestor. The clonal relationships of 270 *S. mutans* isolated from Thai children with three caries statuses (CF, LC, and HC) are shown in Fig. 2. We aimed to find specific clone(s) related to the dental caries status of the host. However, MLST indicated no specific CC related to caries-free status. Based on phenotypic properties examined, *S. mutans* from individuals in the CF group possessed biofilm formation, acidogenic, and aciduric properties not less than those from the LC and HC groups. Therefore, it is reasonable to suggest that the presence of *S. mutans* alone does not predict caries occurrence, as its impact is significantly influenced by host factors and dietary habits. According to the ecological plaque hypothesis, ecological stress, such as frequent exposure to a low pH from sugar fermentation, is essential for the shift from healthy to disease status [53]. Even when we added data on *S. mutans* from the Oral *Streptococcus* PubMLST database into the analysis, the findings confirmed no caries-free related clone in the *S. mutans* population.

Other genotyping methods performed to differentiate *S. mutans* derived from subjects with different caries statuses were gel-based genotyping techniques, such as arbitrary-primed PCR [16], chromosomal DNA fingerprinting with a restriction endonuclease [54], and pulsed-field gel electrophoresis [55]. In keeping with our results, no specific cluster of *S. mutans* associated with caries-free status was found in these studies. In addition, some studies aimed to identify parts of genomes specific to *S. mutans* strains from children with a high number of caries, compared with strains from those with a low number of caries or caries-free children. No differences in the distributions of putative virulence genes, genomic islands, or insertion sequences were found between *S. mutans* strains from children with severe early childhood caries and those isolated from caries-free children [54]. Similarly, comparative genome hybridization using microarray technology and whole genome comparison by in silico genome subtraction failed to indicate any genetic loci of *S. mutans* consistently associated with caries status [15, 56].

Esberg et al. [44] have linked the detection of some surface protein-encoding genes of *S. mutans*, i.e., adhesin SpaP variant B and collagen-binding protein (Cnm), with increased baseline caries and prospective caries development. Our study found two CCs of *S. mutans* with collagen-binding ability (Groups 2 and 13). However, not all *S. mutans* in these two CCs were from caries-affected hosts; consequently, there was no relatedness between clones of collagen-binding *S. mutans* and caries status. Although MLST indicated that seven CCs contained *S. mutans* only from children having dental caries (Groups 3, 10, 11, 13, 14, 15, and 19), *S. mutans* strains in these clones did not show higher abilities to form biofilm, bind to type I collagen, or produce/tolerate acid than those in the CCs from both caries-affected and caries-free children. On adding *S. mutans* from the Oral *Streptococcus* PubMLST database into the analysis, Table 4 shows that Group 10 changed to include *S. mutans* from both caries-free and caries-affected subjects. The other six CCs remained as having no *S. mutans* from caries-free individuals, while 10 more CCs of *S. mutans* from caries-affected hosts only were detected (Groups 20, 21, 24, 27, 28, 29, 31, 32, 33, and 34). However, the number of members in the CCs lacking strains from caries-free subjects was mostly two STs, which is insufficient to be regarded as potential disease-related clones. Because of the efficiency and relatively low cost of modern sequencing technology, standard MLST (7–8 loci) has been expanded to include analysis of more genes, for example, ribosomal MLST (rMLST, 53 loci), core-genome MLST (cgMLST, > 500 loci), and whole-genome MLST (wgMLST, all loci) [57]. Recently, a cgMLST method analyzing 594 core genes was developed for *S. mutans* and showed more discriminatory power than the traditional MLST method [39]. The data for *S. mutans* studied by this cgMLST were also deposited into the Oral *Streptococcus* PubMLST database. Further accumulation of such MLST data and strain analysis might improve understanding of the *S. mutans* population.

## Conclusions

Overall, children with carious lesions showed a higher detection rate and a higher number of *S. mutans* in saliva than those without caries. We found no difference in acid production or acid tolerance in *S. mutans* isolates from Thai children with different caries statuses. Although *S. mutans* from children with high severity of dental caries showed the lowest biofilm formation ability, they had the highest detection rate of collagen-binding isolates. *S. mutans* isolates from caries-free hosts could adhere as well as produce and tolerate acid not less than that of isolates from caries-affected individuals. Genotyping by MLST did not reveal any *S. mutans* clones specific to caries-free individuals. It showed clones containing only *S. mutans* strains from individuals with caries, but the properties of these strains did not differ from those in clones from both caries-free and caries-affected children. Thus, the clonal relationships of *S. mutans* indicated by MLST were not associated with the status of dental caries in the host.

## Supplementary Information

Additional file 1: Table S1 Primers employed in MLST of *S. mutans*.

Additional file 2: Table S2 Allelic profiles and STs of 115 *S. mutans* strains from the Oral Streptococcus PubMLST database.

Additional file 3: Table S3 Allelic profiles and STs of 270 *S. mutans* isolated from the kindergarten children in this study.

Additional file 4: Fig. S1 Acid production by *S. mutans* from Thai children. The comparison was based on caries status: caries-free (CF; *n* = 90 *S*. *mutans* isolates), low severity of caries (LC; *n* = 90), and high severity of caries (HC; *n* = 90). The data shown represent the median values of each group. No significant difference in pH values was observed at any time point.

## Data Availability

All data generated or analyzed during this study are included in the article and its supplementary files. All nucleotide sequences determined in the process of MLST were registered in the GenBank database (accession numbers OQ809072–OQ809341 for *tkt*; OQ829640–OQ829909 for *glnA*; OQ829910–OQ830179 for *gltA*; OQ830180–OQ830449 for *glk*; OQ866634–OQ866903 for *aroE*; OQ938982–OQ939251 for *murI*; OQ939252–OQ939521 for *lepC*; and OQ972993–OQ973262 for *gyrA*). Further enquiries can be directed to the corresponding author.

## Abbreviations

CC: Clonal complex
CF: Caries-free
HC: High severity of caries
LC: Low severity of caries
MLST: Multilocus sequence typing
ST: Sequence type
