# Supplementary material for: Multilocus sequence typing and phenotypic properties of Streptococcus mutans from Thai children with different caries statuses
Source: BMC Oral Health. 2024 Sep 11;24:1063. doi: 10.1186/s12903-024-04759-9 (PMC11391724; doi:10.1186/s12903-024-04759-9)
Supplement: Supplementary file 1 — Additional file 1: Table S1 Primers employed in MLST of S. mutans. [file 12903_2024_4759_MOESM1_ESM.docx]

**Table S1.** Primers employed in MLST of *S. mutans*

| **Gene** | **Primer** | **Sequence**  **(5' to 3')** | **Size of**  **amplicon**  **(bp)** | **Size of**  **sequence fragment**  **(bp)^a^** |
| --- | --- | --- | --- | --- |
| transketolase | *tkt*/F | CAG ATT TAT CGG TTA ATG CCA TTC G | 751 | 432 |
|  | *tkt*/R | TTA GTT GGA GCA CCG TAG CC |  |  |
| glutamine synthetase type I | *glnA*/F | ACA AAG CGA TGT TTG ATG GCT | 631 | 462 |
|  | *glnA*/R | GCG TTC TTA CCA TCA CTG CC |  |  |
| glutamate synthetase | *gltA*/F | TTG AGA CAG ATG CCT GTG GG | 564 | 387 |
|  | *gltA*/R | AAG CAT GCA GCA TTC CCT TA |  |  |
| glucose kinase | *glk*/F | AGG GAT TGA TCT TGG TGG AAC A | 585 | 402 |
|  | *glk*/R | AAA TGA CGT GCA ACA CGG AC |  |  |
| shikimate 5-dehydrogenase | *aroE*/F | ATG CCT TAC AAG CAG GCA GT | 642 | 397 |
|  | *aroE*/R | AGC CTG CCA GAT TTC CTG AC |  |  |
| glutamate racemase | *murI*/F | GAC CTA TTG GTT TTT TAG ACT CCG | 520 | 423 |
|  | *murI*/R | TCA ATT TTC CCC ACC AGA GGA |  |  |
| signal peptidase I | *lepC*/F | AGA ATG GGG CCT TTT CTT GGT C | 536 | 418 |
|  | *lepC*/R | GCC AAA AGC GGA ATT TAA CTT CAC C |  |  |
| DNA gyrase A subunit | *gyrA*/F | TCG GGC TCT TCC AGA TGT TC | 605 | 435 |
|  | *gyrA*/R | AGG CGC GAT GTA TAC CCG AT |  |  |

^a^ Sizes and locations of sequence fragments are based on the study of Nakano et al., [12] and the oral *Streptococcus* MLST database.
