# Supplementary material for: Multilocus sequence typing and phenotypic properties of Streptococcus mutans from Thai children with different caries statuses
Source: BMC Oral Health. 2024 Sep 11;24:1063. doi: 10.1186/s12903-024-04759-9 (PMC11391724; doi:10.1186/s12903-024-04759-9)
Supplement: Supplementary file 2 — Additional file 2: Table S2 Allelic profiles and STs of 115 S. mutans strains from the Oral Streptococcus PubMLST database. [file 12903_2024_4759_MOESM2_ESM.docx]

**Table S2** Allelic profiles and STs of 115 *S. mutans* strains from the Oral *Streptococcus* PubMLST database

| ID^a^ | ST | Allelic profile | | | | | | | | Caries status | Country of origin |
| --- | --- | --- | --- | --- | --- | --- | --- | --- | --- | --- | --- |
|  |  | *tkt* | *glnA* | *gltA* | *glk* | *aroE* | *murI* | *lepC* | *gyrA* |  |  |
| 543 | 148 | 1 | 1 | 1 | 1 | 11 | 3 | 3 | 6 | Caries | USA |
| 544 | 149 | 1 | 1 | 5 | 8 | 26 | 3 | 1 | 1 | Caries | USA |
| 545 | 150 | 1 | 1 | 1 | 1 | 2 | 3 | 1 | 1 | Caries | USA |
| 546 | 151 | 19 | 2 | 10 | 8 | 8 | 3 | 5 | 1 | Caries | USA |
| 547 | 152 | 2 | 3 | 25 | 8 | 11 | 2 | 5 | 4 | Caries | USA |
| 548 | 153 | 1 | 1 | 31 | 1 | 27 | 2 | 11 | 1 | Caries | USA |
| 549 | 154 | 16 | 2 | 15 | 8 | 1 | 2 | 11 | 3 | Caries | USA |
| 550 | 155 | 1 | 2 | 15 | 8 | 1 | 11 | 11 | 1 | Caries | USA |
| 551 | 156 | 3 | 8 | 15 | 8 | 2 | 5 | 21 | 1 | Caries | USA |
| 552 | 157 | 2 | 2 | 4 | 5 | 4 | 5 | 1 | 1 | Caries | USA |
| 553 | 158 | 20 | 2 | 24 | 8 | 4 | 3 | 1 | 1 | Caries | USA |
| 554 | 159 | 18 | 23 | 1 | 23 | 14 | 21 | 11 | 21 | Caries-free | USA |
| 555 | 160 | 1 | 1 | 5 | 8 | 4 | 3 | 31 | 1 | Caries | USA |
| 556 | 161 | 1 | 1 | 5 | 24 | 4 | 3 | 31 | 1 | Caries | USA |
| 557 | 162 | 1 | 3 | 15 | 8 | 4 | 2 | 19 | 1 | Caries | USA |
| 558 | 163 | 1 | 1 | 32 | 8 | 4 | 19 | 5 | 4 | Caries | USA |
| 559 | 164 | 1 | 8 | 33 | 8 | 4 | 3 | 11 | 1 | Caries-free | USA |
| 560 | 165 | 18 | 3 | 1 | 1 | 2 | 3 | 32 | 1 | Caries-free | USA |
| 561 | 166 | 3 | 24 | 5 | 25 | 1 | 1 | 30 | 15 | Caries | USA |
| 562 | 167 | 1 | 16 | 23 | 1 | 11 | 3 | 1 | 15 | Caries | USA |
| 563 | 168 | 3 | 2 | 15 | 1 | 28 | 3 | 11 | 1 | Caries | USA |
| 564 | 169 | 1 | 1 | 1 | 1 | 2 | 3 | 11 | 1 | Caries-free | USA |
| 565 | 170 | 3 | 10 | 15 | 24 | 21 | 2 | 1 | 1 | Caries | USA |
| 566 | 171 | 3 | 10 | 15 | 8 | 21 | 2 | 1 | 1 | Caries | USA |
| 567 | 172 | 21 | 3 | 20 | 4 | 27 | 5 | 1 | 1 | Caries | USA |
| 568 | 173 | 3 | 21 | 1 | 21 | 2 | 4 | 11 | 19 | Caries | USA |
| 599 | 175 | 1 | 2 | 1 | 8 | 4 | 3 | 1 | 1 | Caries | USA |
| 600 | 176 | 1 | 1 | 1 | 23 | 1 | 1 | 1 | 1 | Caries | USA |
| 601 | 177 | 1 | 25 | 31 | 3 | 1 | 1 | 1 | 1 | Caries | USA |
| 602 | 178 | 1 | 1 | 31 | 8 | 4 | 5 | 1 | 6 | Caries | USA |
| 603 | 179 | 1 | 2 | 15 | 3 | 1 | 11 | 11 | 1 | Caries-free | USA |
| 604 | 180 | 3 | 8 | 15 | 8 | 29 | 5 | 21 | 1 | Caries | USA |
| 605 | 181 | 2 | 2 | 4 | 5 | 4 | 22 | 30 | 1 | Caries | USA |
| 606 | 182 | 2 | 2 | 5 | 1 | 27 | 23 | 1 | 1 | Caries | USA |
| 607 | 183 | 6 | 3 | 20 | 4 | 4 | 5 | 33 | 4 | Caries | USA |
| 609 | 185 | 1 | 2 | 1 | 1 | 19 | 3 | 24 | 1 | Caries | USA |
| 610 | 186 | 1 | 2 | 34 | 4 | 2 | 24 | 1 | 21 | Caries | USA |
| 611 | 187 | 22 | 2 | 31 | 4 | 4 | 3 | 11 | 1 | Caries | USA |
| 612 | 188 | 3 | 2 | 1 | 23 | 30 | 3 | 34 | 1 | Caries | USA |
| 613 | 189 | 2 | 3 | 20 | 4 | 5 | 5 | 1 | 22 | Caries | USA |
| 614 | 190 | 23 | 8 | 15 | 8 | 2 | 5 | 21 | 1 | Caries | USA |
| 615 | 191 | 1 | 1 | 8 | 8 | 31 | 3 | 1 | 1 | Caries | USA |
| 619 | 195 | 1 | 1 | 1 | 13 | 2 | 1 | 1 | 1 | Caries | USA |
| 620 | 196 | 1 | 1 | 1 | 5 | 2 | 1 | 1 | 1 | Caries | USA |
| 622 | 198 | 1 | 2 | 7 | 1 | 14 | 7 | 19 | 1 | Caries | USA |
| 623 | 199 | 1 | 2 | 15 | 3 | 1 | 3 | 11 | 1 | Caries | USA |
| 626 | 202 | 3 | 2 | 1 | 1 | 4 | 11 | 1 | 1 | Caries-free | USA |
| 627 | 203 | 1 | 2 | 15 | 3 | 1 | 3 | 1 | 4 | Caries | USA |
|  |  |  | | | | | | | |  |  |
|  |  |  | | | | | | | |  |  |
|  |  |  | | | | | | | |  |  |
|  |  |  | | | | | | | |  |  |
| ID^a^ | ST | Allelic profile | | | | | | | | Caries status | Country of origin |
|  |  | *tkt* | *glnA* | *gltA* | *glk* | *aroE* | *murI* | *lepC* | *gyrA* |  |  |
| 628 | 204 | 18 | 23 | 35 | 23 | 14 | 21 | 11 | 21 | Caries | USA |
| 629 | 205 | 1 | 3 | 15 | 3 | 4 | 2 | 19 | 1 | Caries-free | USA |
| 630 | 206 | 1 | 1 | 32 | 3 | 4 | 19 | 5 | 4 | Caries | USA |
| 631 | 207 | 20 | 2 | 24 | 3 | 4 | 3 | 1 | 1 | Caries-free | USA |
| 632 | 208 | 19 | 2 | 10 | 3 | 8 | 3 | 5 | 1 | Caries | USA |
| 633 | 209 | 2 | 2 | 4 | 3 | 4 | 25 | 1 | 4 | Caries | USA |
| 634 | 210 | 1 | 2 | 5 | 3 | 14 | 3 | 1 | 23 | Caries | USA |
| 635 | 211 | 1 | 16 | 8 | 1 | 2 | 3 | 1 | 1 | Caries | USA |
| 636 | 212 | 14 | 2 | 4 | 1 | 2 | 3 | 1 | 24 | Caries | USA |
| 736 | 231 | 3 | 3 | 39 | 4 | 34 | 27 | 1 | 1 | Caries | China |
| 737 | 232 | 27 | 9 | 40 | 1 | 14 | 3 | 1 | 1 | Caries | China |
| 738 | 233 | 3 | 9 | 41 | 26 | 34 | 2 | 1 | 1 | Caries | China |
| 739 | 265 | 31 | 5 | 51 | 1 | 23 | 2 | 1 | 1 | Caries | China |
| 740 | 235 | 28 | 2 | 43 | 3 | 8 | 28 | 5 | 1 | Caries | China |
| 741 | 236 | 3 | 8 | 44 | 8 | 2 | 2 | 36 | 1 | Caries | China |
| 742 | 237 | 1 | 1 | 1 | 27 | 11 | 19 | 1 | 1 | Caries | China |
| 743 | 269 | 3 | 9 | 41 | 4 | 14 | 3 | 11 | 1 | Caries | China |
| 744 | 239 | 3 | 9 | 41 | 1 | 34 | 27 | 1 | 1 | Caries | China |
| 745 | 240 | 2 | 3 | 45 | 3 | 11 | 2 | 5 | 4 | Caries | China |
| 746 | 241 | 29 | 5 | 46 | 1 | 2 | 3 | 1 | 1 | Caries | China |
| 747 | 238 | 3 | 1 | 1 | 28 | 14 | 3 | 11 | 1 | Caries | China |
| 748 | 243 | 28 | 2 | 44 | 1 | 7 | 27 | 1 | 1 | Caries | China |
| 749 | 244 | 1 | 2 | 47 | 8 | 1 | 29 | 1 | 3 | Caries | China |
| 750 | 245 | 1 | 2 | 44 | 1 | 7 | 27 | 1 | 25 | Caries | China |
| 751 | 246 | 6 | 3 | 48 | 4 | 27 | 5 | 1 | 26 | Caries | China |
| 752 | 247 | 1 | 1 | 39 | 3 | 1 | 14 | 1 | 1 | Caries | China |
| 753 | 248 | 2 | 2 | 44 | 29 | 2 | 3 | 37 | 1 | Caries | China |
| 754 | 247 | 1 | 1 | 39 | 3 | 1 | 14 | 1 | 1 | Caries | China |
| 755 | 250 | 27 | 9 | 41 | 4 | 14 | 3 | 11 | 1 | Caries | China |
| 756 | 251 | 1 | 2 | 1 | 3 | 2 | 27 | 38 | 3 | Caries | China |
| 757 | 252 | 28 | 2 | 49 | 3 | 8 | 28 | 5 | 1 | Caries | China |
| 758 | 243 | 28 | 2 | 44 | 1 | 7 | 27 | 1 | 1 | Caries | China |
| 759 | 254 | 30 | 2 | 49 | 3 | 8 | 28 | 5 | 1 | Caries | China |
| 760 | 238 | 3 | 1 | 1 | 28 | 14 | 3 | 11 | 1 | Caries | China |
| 761 | 256 | 1 | 9 | 41 | 13 | 4 | 3 | 11 | 1 | Caries | China |
| 762 | 257 | 3 | 2 | 42 | 1 | 2 | 30 | 3 | 1 | Caries | China |
| 763 | 258 | 1 | 1 | 1 | 20 | 22 | 27 | 1 | 1 | Caries | China |
| 764 | 247 | 1 | 1 | 39 | 3 | 1 | 14 | 1 | 1 | Caries | China |
| 765 | 260 | 3 | 2 | 50 | 1 | 18 | 3 | 11 | 1 | Caries | China |
| 766 | 246 | 6 | 3 | 48 | 4 | 27 | 5 | 1 | 26 | Caries | China |
| 767 | 293 | 3 | 2 | 42 | 1 | 21 | 11 | 24 | 1 | Caries-free | China |
| 769 | 295 | 32 | 1 | 1 | 1 | 22 | 3 | 1 | 1 | Caries-free | China |
| 771 | 297 | 33 | 1 | 1 | 20 | 4 | 19 | 1 | 1 | Caries-free | China |
| 773 | 299 | 4 | 1 | 1 | 2 | 2 | 3 | 1 | 1 | Caries-free | China |
| 775 | 301 | 27 | 2 | 1 | 3 | 7 | 20 | 1 | 1 | Caries-free | China |
| 777 | 303 | 1 | 3 | 39 | 1 | 4 | 3 | 1 | 1 | Caries-free | China |
| 779 | 257 | 3 | 2 | 42 | 1 | 2 | 30 | 3 | 1 | Caries-free | China |
| 781 | 307 | 1 | 14 | 15 | 8 | 35 | 3 | 1 | 1 | Caries-free | China |
| 783 | 236 | 3 | 8 | 44 | 8 | 2 | 2 | 36 | 1 | Caries-free | China |
| 785 | 261 | 1 | 3 | 1 | 3 | 1 | 11 | 11 | 26 | Caries | Iran |
| 786 | 242 | 28 | 1 | 1 | 1 | 4 | 14 | 16 | 1 | Caries | Iran |
|  |  |  | | | | | | | |  |  |
|  |  |  | | | | | | | |  |  |
|  |  |  | | | | | | | |  |  |
| ID^a^ | ST | Allelic profile | | | | | | | | Caries status | Country of origin |
|  |  | *tkt* | *glnA* | *gltA* | *glk* | *aroE* | *murI* | *lepC* | *gyrA* |  |  |
| 787 | 253 | 28 | 2 | 1 | 22 | 1 | 7 | 16 | 1 | Caries | Iran |
| 788 | 263 | 28 | 3 | 1 | 3 | 1 | 3 | 1 | 1 | Caries | Iran |
| 789 | 264 | 14 | 1 | 1 | 1 | 11 | 3 | 1 | 26 | Caries | Iran |
| 790 | 262 | 31 | 1 | 1 | 1 | 1 | 19 | 1 | 19 | Caries | Afghanistan |
| 791 | 255 | 28 | 2 | 1 | 1 | 1 | 3 | 19 | 1 | Caries | Afghanistan |
| 792 | 259 | 1 | 22 | 5 | 1 | 1 | 1 | 1 | 26 | Caries | Afghanistan |
| 2079 | 266 | 1 | 1 | 53 | 13 | 1 | 1 | 39 | 1 | Caries | Sweden |
| 2080 | 267 | 2 | 2 | 1 | 3 | 4 | 25 | 1 | 4 | Caries | Sweden |
| 2081 | 268 | 4 | 26 | 1 | 3 | 2 | 27 | 1 | 3 | Caries-free | Sweden |
| 2082 | 270 | 4 | 1 | 52 | 2 | 4 | 5 | 32 | 1 | Caries | Sweden |
| 2083 | 271 | 1 | 3 | 39 | 1 | 25 | 3 | 1 | 1 | Caries | Sweden |
| 2084 | 272 | 1 | 2 | 44 | 3 | 1 | 11 | 1 | 3 | Caries | Sweden |
| 2085 | 273 | 34 | 3 | 48 | 4 | 5 | 5 | 1 | 4 | Caries | Sweden |
| 2086 | 274 | 25 | 2 | 1 | 4 | 4 | 2 | 32 | 1 | Caries | Sweden |
| 2087 | 275 | 1 | 3 | 39 | 1 | 36 | 10 | 3 | 1 | Caries | Sweden |
| 2088 | 276 | 6 | 3 | 48 | 4 | 4 | 5 | 1 | 4 | Caries | Sweden |

^a^ ID as presented in the Oral *Streptococcus* PubMLST database (http://pubmlst.org/oralstrep/). ST, sequence type.
