# Supplementary material for: Multilocus sequence typing and phenotypic properties of Streptococcus mutans from Thai children with different caries statuses
Source: BMC Oral Health. 2024 Sep 11;24:1063. doi: 10.1186/s12903-024-04759-9 (PMC11391724; doi:10.1186/s12903-024-04759-9)
Supplement: Supplementary file 3 — Additional file 3: Table S3 Allelic profiles and STs of 270 S. mutans isolated from the kindergarten children in this study. [file 12903_2024_4759_MOESM3_ESM.docx]

**Table S3** Allelic profiles and STs of 270 *S. mutans* isolated from the kindergarten children in this study

| ST | Allelic profile^a^ | | | | | | | | No. of strains detected |
| --- | --- | --- | --- | --- | --- | --- | --- | --- | --- |
|  | *tkt* | *glnA* | *gltA* | *glk* | *aroE* | *murI* | *lepC* | *gyrA* |  |
| 2 | 1 | 1 | 1 | 13 | 1 | 1 | 1 | 1 | 14 |
| 252 | 28 | 2 | 49 | 3 | 8 | 28 | 5 | 1 | 13 |
| 247 | 1 | 1 | 39 | 3 | 1 | 14 | 1 | 1 | 8 |
| 309 | 3 | 3 | 39 | 20 | 4 | 2 | 1 | 10 | 7 |
| 312 | 2 | 2 | **53** | 3 | 4 | 25 | 1 | 4 | 7 |
| 63 | 4 | 1 | 1 | 3 | 2 | 3 | 1 | 1 | 6 |
| 238 | 3 | 1 | 1 | 28 | 14 | 3 | 11 | 1 | 6 |
| 276 | 6 | 3 | 48 | 4 | 4 | 5 | 1 | 4 | 6 |
| 279 | 3 | 2 | 1 | 6 | 18 | 27 | 24 | **27** | 6 |
| 285 | 20 | 2 | **56** | 3 | 4 | 28 | 1 | 1 | 6 |
| 286 | 1 | 1 | 1 | 20 | 22 | 19 | 1 | 1 | 6 |
| 299 | 4 | 1 | 1 | 2 | 2 | 3 | 1 | 1 | 6 |
| 316 | 3 | 1 | 1 | 28 | 18 | 3 | 28 | 1 | 6 |
| 322 | 2 | 2 | **53** | 5 | 4 | 22 | 30 | 1 | 6 |
| 342 | **37** | 2 | **55** | 3 | 16 | 7 | 1 | 1 | 6 |
| 240 | 2 | 3 | 45 | 3 | 11 | 2 | 5 | 4 | 5 |
| 282 | **35** | 2 | 44 | 8 | 4 | **31** | 3 | 1 | 5 |
| 292 | 3 | 14 | **57** | 8 | 2 | 15 | 19 | 1 | 5 |
| 351 | 14 | 2 | **61** | 1 | 2 | 3 | 1 | 1 | 5 |
| 294 | 1 | 2 | 42 | 3 | 16 | 7 | 1 | 1 | 4 |
| 304 | 1 | 2 | **55** | 3 | 16 | 7 | 1 | 1 | 4 |
| 321 | 1 | 1 | 1 | 1 | 18 | 3 | 3 | 1 | 4 |
| 236 | 3 | 8 | 44 | 8 | 2 | 2 | 36 | 1 | 3 |
| 278 | 3 | 2 | 44 | 3 | 1 | 11 | 1 | 3 | 3 |
| 280 | 3 | 5 | 41 | 6 | 4 | 20 | 11 | 1 | 3 |
| 281 | 15 | 2 | 41 | 1 | 4 | 27 | 3 | 11 | 3 |
| 283 | 3 | **27** | 41 | 6 | 2 | 20 | 11 | 1 | 3 |
| 287 | 3 | 2 | 42 | 3 | 2 | 11 | 3 | 1 | 3 |
| 291 | 3 | 10 | 44 | 3 | **41** | 2 | 1 | 1 | 3 |
| 300 | 3 | 5 | 41 | 17 | 20 | 15 | 36 | 1 | 3 |
| 306 | 4 | 1 | **52** | 1 | 2 | 5 | 1 | 1 | 3 |
| 308 | 16 | 2 | 44 | 3 | 1 | 11 | 1 | 3 | 3 |
| 311 | 2 | 9 | 41 | 1 | 11 | 19 | 1 | 1 | 3 |
| 313 | **36** | 5 | 41 | 6 | 18 | 11 | 19 | 1 | 3 |
| 317 | 1 | 2 | 1 | 3 | 2 | 27 | 1 | 1 | 3 |
| 319 | 3 | 5 | 41 | 1 | 2 | 5 | 3 | 1 | 3 |
| 320 | 1 | 2 | 1 | 3 | 2 | 27 | 1 | 3 | 3 |
| 323 | 1 | 2 | **53** | 8 | **37** | 3 | **42** | 1 | 3 |
| 325 | 28 | 2 | 49 | 3 | 3 | 28 | 5 | 1 | 3 |
| 326 | 6 | 3 | 48 | 4 | 5 | 5 | 1 | 4 | 3 |
| 327 | 3 | 14 | **57** | 6 | 17 | **32** | 11 | 1 | 3 |
| 330 | **39** | 1 | 1 | 3 | **38** | 11 | 30 | 1 | 3 |
| 331 | 1 | 14 | 47 | **31** | **39** | 7 | **43** | 1 | 3 |
| 336 | **37** | 3 | 42 | 8 | 11 | 2 | 9 | 3 | 3 |
| 343 | 4 | 1 | 1 | 2 | 4 | 3 | 1 | 1 | 3 |
| 345 | 3 | 5 | 41 | 1 | 2 | 3 | 3 | 1 | 3 |
| 346 | 2 | 2 | **53** | 5 | 4 | 5 | 1 | 4 | 3 |
| 347 | **36** | 5 | **62** | 6 | 18 | 11 | 19 | 1 | 3 |
|  |  |  |  |  |  |  |  |  |  |
|  |  |  |  |  |  |  |  |  |  |
|  |  |  |  |  |  |  |  |  |  |
|  |  |  |  |  |  |  |  |  |  |
| ST | Allelic profile^a^ | | | | | | | | No. of strains detected |
|  | *tkt* | *glnA* | *gltA* | *glk* | *aroE* | *murI* | *lepC* | *gyrA* |  |
| 348 | **37** | 16 | **60** | 4 | 2 | 18 | 22 | 15 | 3 |
| 271 | 1 | 3 | 39 | 1 | 25 | 3 | 1 | 1 | 2 |
| 288 | 3 | 21 | 42 | 21 | 2 | 3 | 40 | 19 | 2 |
| 296 | 3 | 9 | 41 | 1 | 2 | 3 | 3 | 10 | 2 |
| 302 | **36** | **27** | **54** | 6 | 4 | 11 | 19 | 1 | 2 |
| 314 | **38** | 5 | 41 | 6 | 2 | 3 | 1 | 1 | 2 |
| 324 | **37** | 2 | **55** | 3 | 11 | 7 | 1 | 1 | 2 |
| 328 | 3 | 9 | 43 | 4 | 2 | 3 | 3 | 10 | 2 |
| 329 | 2 | 2 | 1 | 1 | 27 | 23 | 1 | 1 | 2 |
| 332 | 3 | 14 | **57** | 8 | 2 | 15 | **44** | 1 | 2 |
| 333 | **36** | 5 | 41 | 6 | **40** | 11 | 19 | 1 | 2 |
| 338 | 3 | 2 | 1 | 6 | 4 | 27 | 24 | **27** | 2 |
| 340 | 1 | 1 | 1 | 20 | 11 | 19 | 1 | 1 | 2 |
| 341 | 2 | 5 | 44 | 3 | 18 | 3 | 1 | 1 | 2 |
| 344 | 1 | 1 | 39 | 3 | 1 | 14 | 1 | **28** | 2 |
| 349 | 3 | 2 | 1 | 8 | 2 | 3 | 3 | 3 | 2 |
| 352 | 3 | 14 | 42 | 17 | 20 | 15 | 36 | 1 | 2 |
| 131 | 3 | 1 | 1 | 4 | 2 | 19 | 1 | 15 | 1 |
| 270 | 4 | 1 | **52** | 2 | 4 | 5 | 32 | 1 | 1 |
| 277 | 6 | 1 | **58** | 4 | 5 | 5 | 1 | 4 | 1 |
| 284 | **37** | 19 | **59** | 3 | 2 | 18 | 1 | 15 | 1 |
| 289 | 29 | 2 | 50 | **30** | 2 | 3 | 1 | 1 | 1 |
| 290 | 1 | 1 | 1 | 3 | 2 | 27 | 1 | 3 | 1 |
| 298 | **36** | 5 | 41 | 6 | 2 | 3 | 11 | 1 | 1 |
| 305 | **36** | 5 | 41 | 20 | 4 | 2 | 11 | 10 | 1 |
| 310 | **37** | 2 | **55** | 3 | 16 | 7 | **41** | 1 | 1 |
| 315 | 6 | 3 | 48 | 4 | 4 | 5 | 5 | 4 | 1 |
| 318 | 6 | 2 | **56** | 3 | 4 | 28 | 1 | 1 | 1 |
| 334 | 2 | 2 | 41 | 1 | 2 | 5 | 27 | 1 | 1 |
| 335 | 3 | 5 | 41 | 6 | 2 | 5 | 3 | 1 | 1 |
| 337 | 1 | 3 | 39 | 4 | 2 | 27 | 1 | 1 | 1 |
| 339 | 1 | 5 | **63** | 3 | 4 | 5 | 19 | 1 | 1 |
| 350 | 14 | 2 | **61** | 1 | 2 | 3 | 1 | **29** | 1 |

ST, sequence type. ^a^ New alleles identified in this study are indicated in bold.
