# Supplementary material for: Multilocus sequence typing and phenotypic properties of Streptococcus mutans from Thai children with different caries statuses
Source: BMC Oral Health. 2024 Sep 11;24:1063. doi: 10.1186/s12903-024-04759-9 (PMC11391724; doi:10.1186/s12903-024-04759-9)
Supplement: Supplementary file 4 — Additional file 4: Fig. S1 Acid production by S. mutans from Thai children. The comparison was based on caries status: caries-free (CF; n = 90 S. mutans isolates), low severity of caries (LC; n = 90), and high severity of caries (HC; n = 90). The data shown represent the median values of each group. No significant difference in pH values was observed at any time point. [file 12903_2024_4759_MOESM4_ESM.docx]

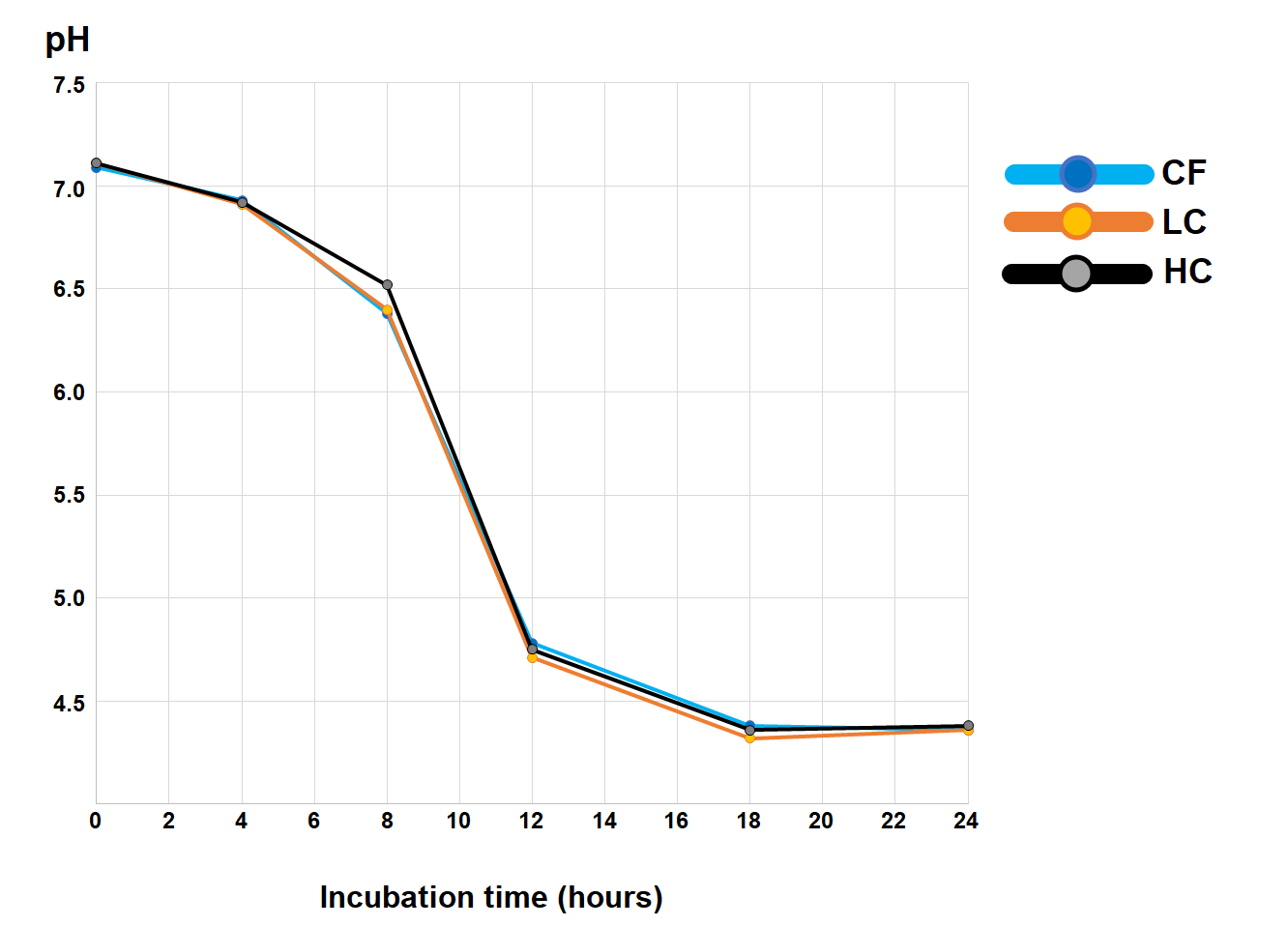


**Fig. S1** Acid production by *S. mutans* from Thai children. The comparison was based on caries status: caries-free (CF; *n =* 90 *S. mutans* isolates), low severity of caries (LC; *n =* 90), and high severity of caries (HC; *n =* 90). The data shown represent the median values of each group. No significant difference in pH values was observed at any time point.

**Fig. S1**
